# Sexual and regional differences in the microbiome and functional metagenome of the lone star tick, *Amblyomma americanum*

**DOI:** 10.1186/s42523-025-00498-6

**Published:** 2025-12-06

**Authors:** Peter Briggs, Lawson Trimmell, Leah T. Stiemsma, Javier Monzón

**Affiliations:** Natural Science Division, Pepperdine University, Pacific Coast Highway, Malibu, CA 24255 USA

**Keywords:** *Amblyomma americanum*, *Coxiella*, Functional metagenome, Lone star tick, Microbiome

## Abstract

**Background:**

Ticks are important vectors of pathogens that cause disease in humans and domestic and wild animals. Understanding how microbes within ticks interact among themselves and with their tick host is a significant step in controlling diseases vectored by ticks and other arthropods. We used Illumina sequencing of the 16S rRNA bacterial gene to characterize the diversity and composition of the microbiome of the lone star tick (*Amblyomma americanum*), an aggressive tick in the United States that is expanding its geographic range both westward and northward. Specifically, we examined differences in microbial community structure and metabolic pathways between two regions at the boundary of the lone star tick’s current range, between sexes, and between wild and laboratory-reared tick populations.

**Results:**

Geographic region and sex strongly influence alpha diversity, beta diversity, the relative abundance of particular taxa, and the prevalence of pathogens. Analyses of phylogenetic structure suggest that bacterial community assembly is shaped not by competitive exclusion, but by environmental filtering related to tick physiology—particularly in females. Additionally, we show that distinct taxonomic profiles of the tick microbiome lead to distinct functional profiles between the sexes, highlighting important vitamin and heme metabolic pathways that are significantly more abundant in the metagenomes of female ticks.

**Conclusion:**

This study integrates taxonomic profiling and predictive functional metagenomics to elucidate important associations between ticks and their microbes. It is the largest tick microbiome investigation to date based on next-generation sequencing of the 16S rRNA bacterial gene, and it reveals that both sex and geographic region affect various aspects of the microbiome and functional metagenome of *A. americanum.* Discovering the mechanisms through which microbes help ticks can make dysbiosis a potential strategy for tick control.

**Supplementary Information:**

The online version contains supplementary material available at 10.1186/s42523-025-00498-6.

## Background

Worldwide, ticks are ranked second after mosquitoes as vectors of human pathogens, but are ranked first as the most important vectors of pathogens of domestic and wild animals [1]. Ticks are the primary arthropod vectors of human pathogens in North America and Europe. In the United States, the incidence of human tick-borne diseases greatly outnumbers the incidence of insect-borne diseases transmitted by mosquitoes, flies, and fleas combined [2]. Although several tick-borne diseases are caused by eukaryotic microorganisms (e.g. *Babesia*, *Theileria, Cytauxzoon*) and viruses (e.g. Powassan and Heartland viruses), many are caused by bacteria (e.g. *Borrelia*, *Rickettsia*, *Ehrlichia*, *Anaplasma*, *Francisella*) [3–5].

The tick-borne eukaryotic and prokaryotic organisms that cause disease in vertebrates are members of a much more diverse microbial community that inhabits the tick’s internal organs, tissues, and cells [6–8]. As in any ecological community, its constituent species interact in a number of ways that might include competition and mutualism. Understanding how innocuous microbes interact with pathogenic organisms - either in competitive or facilitative ways - is of immense biomedical and veterinary importance; however, these interactions among microbial species in ticks are poorly understood. Furthermore, the habitat of the microbes is itself a living organism whose own physiology, fitness, and vectorial competence are likely affected by the composition of its microbiome [7, 9, 10]. The mechanisms that underlie these effects on tick physiology and fitness are also not well characterized, but are being elucidated by experimental studies of endosymbiont function [11] and omics studies of the functional metagenome of whole tick-associated microbial communities [12–15].

Discovering how microbes within ticks interact among themselves and with their tick host is a significant step in controlling diseases vectored by ticks and other arthropods. For example, certain endosymbionts that are vertically transmitted from mothers to eggs competitively inhibit infection by pathogens within ticks or upregulate the tick’s immune system [16, 17]. Other endosymbionts actually promote the acquisition of pathogens [18, 19]. *Wolbachia*, often present in ticks, is a defensive endosymbiont in insects such as mosquitoes, suggesting a similar effect in ticks [20]. These findings of microbial interactions have applications in vector control, such as the case in which *Wolbachia* was deliberately established in wild *Aedes* mosquito populations to suppress dengue transmission [21]. Hence, non-pathogenic and pathogenic microorganisms are likely to interact in ways that inhibit or facilitate tick-borne pathogen transmission to vertebrate hosts.

The lone star tick (*Amblyomma americanum*) is an aggressive tick found in the eastern half of the United States. Among all ticks that infest humans across the United States, the lone star tick is second only to the blacklegged tick (*Ixodes scapularis*) in biting encounters, but it is the top human-biting tick in the southeastern and Atlantic states [22, 23]. The lone star tick is one of the most important ticks in North America in terms of its medical and veterinary relevance [24, 25]. It is a confirmed competent vector of a variety of pathogenic bacteria that infect humans, including *Ehrlichia chaffeensis* and *Ehrlichia ewingii* which cause ehrlichiosis, and *Francisella tularensis* which causes tularemia. *Rickettsia amblyommatis* is a member of the Spotted Fever Group Rickettsia clade and common in *A. americanum*, although its pathogenicity remains uncertain; most likely, *R. amblyommatis* causes a self-limiting mild febrile illness in mammals and humans [26]. *Borrelia lonestari*, a species in the Relapsing Fever Group Borrelia clade, is associated with the lone star tick as its name suggests. Like *R. amblyommatis*, the pathogenicity of *B. lonestari* remains controversial but this species may represent an emerging tick-borne pathogen [27]. The lone star tick has been expanding its geographic range both westward and northward since the mid-1900s [28, 29]. Its ongoing range expansion and aggressive host-seeking behavior have heightened concern over the potential rise in lone star tick–associated disease cases.

In this study, we used 16S rRNA gene sequencing to characterize the bacterial microbiome of 259 lone star ticks in areas of recent range expansion. Specifically, we sampled three populations along the western expansion front and three populations along the northern expansion front. Additionally, we sampled a laboratory-reared colony population. We predicted that there would be significant differences in microbiome community structure between geographic regions and among sites within regions due to ecological differences. Since previous studies also found important differences between the microbiomes of male and female ticks [30–32] and between the microbiomes of field-collected and laboratory-reared ticks [33–35], we also predicted differences in microbiome community structure between the two sexes and between wild and laboratory-reared populations. Further, we used the 16S rRNA gene sequencing data to predict the functional metagenome of bacterial communities in the lone star tick and examined differences in metabolic pathways between geographic regions and between sexes.

## Methods

### Field collections and samples

**Fig. 1:**
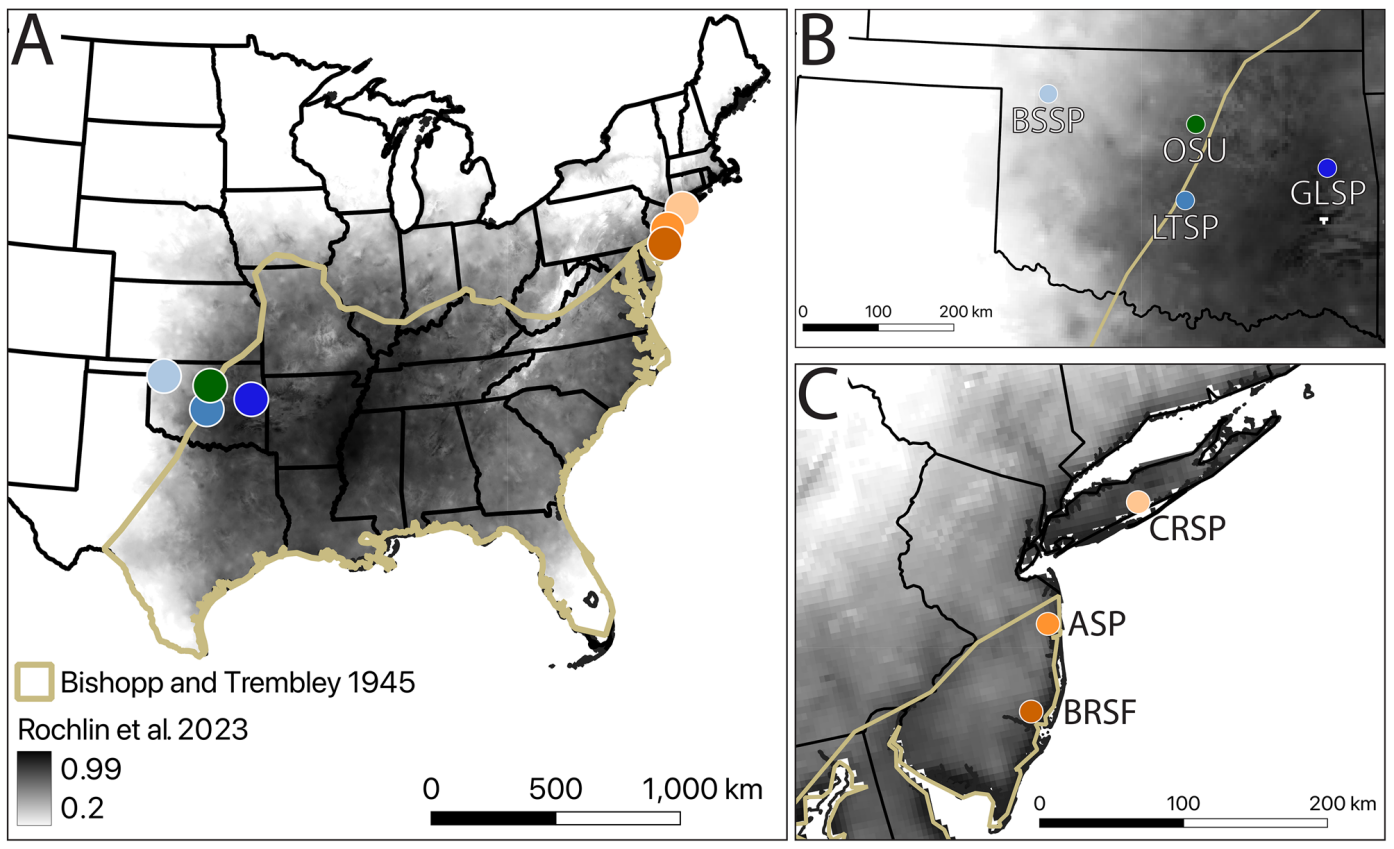
**A**. Geographic range of lone star tick in the United States. Gold = approximate boundaries of historical species distribution, as estimated by Bishopp and Trembley [36]. Gray shading = approximate current species distribution, as modeled by Rochlin et al. [29]. **B**. Western sampling sites in Oklahoma: BSSP = Boiling Springs State Park, LTSP = Lake Thunderbird State Park, GLSP = Greenleaf State Park; OSU = Oklahoma State University Tick Rearing Facility. **C**. Northern sampling sites in New York and New Jersey: CRSP = Connetquot River State Park, ASP = Allaire State Park, BRSF = Bass River State Forest

We collected questing *Amblyomma americanum* from six sites in two regions representing distinct expansion fronts separated by more than 1,800 km (Fig. 1A). On the western expansion front, three sites were spread east to west across Oklahoma (OK): Greenleaf State Park (GLSP) in Muskogee County, Lake Thunderbird State Park (LTSP) in Cleveland County, and Boiling Springs State Park (BSSP) in Woodward County (Fig. 1B). On the northern expansion front, three sites were spread south to north across New Jersey (NJ) and New York (NY): Bass River State Forest (BRSF) in Burlington County, Allaire State Park (ASP) in Monmouth County, and Connetquot River State Park (CRSP) in Suffolk County (Fig. 1C). Details of our collection methods and the ecology of these sites are described by Briggs et al. [2].

We selected 40 adult *A. americanum* ticks per site in a 1:1 sex ratio. The only exception was Allaire State Park (9 males, 31 females) because our field collection of adults was strongly female biased and we only had 9 adult males available for this study. Additionally, we selected 40 adult *A. americanum* ticks in a 1:1 sex ratio from the laboratory-reared colony of Oklahoma State University (OSU) Tick Rearing Facility. Total sample size was 280 ticks.

### DNA extraction, amplification of 16S rRNA gene, and preparation of sequencing libraries

We washed ticks in a sodium hypochlorite (bleach) solution, followed by three sterile water rinses, to remove environmental contaminants from the external surface of the ticks. Sodium hypochlorite is an effective surface decontaminant and recommended for downstream analyses of tick microbiomes [37]. We then bisected washed ticks with sterile disposable scalpels and homogenized for 2 minutes in zirconium bead lysis tubes [38]. We extracted total genomic DNA using the Qiagen DNeasy PowerSoil Pro Kit and quantified DNA concentrations using a Qubit 4 fluorometer. We plated all samples in duplicate in 96-well plates and stored at −20 °C. Every plate contained DNA from a ZymoBIOMICS Microbial Community Standard as a positive control, two extraction blanks as negative controls, and sterile water as another no-template negative control.

We targeted the V4 region of the 16S rRNA gene, widely used for investigation of bacterial diversity [39]. We used primers F515/R806 (TCGTCGGCAGCGTCAGATGTGTATAAGAGACAGGTGCCAGCMGCCGCGGTAA and GTCTCGTGGGCTCGGAGATGTGTATAAGAGACAGGGACTACHVGGGTWTCTAAT), which include overhang sequences that align to the Illumina Nextera XT Index Kit v2 primer sets [40]. The amplification PCR recipe included 12 µl of water, 5 µl of Qiagen AllTaq Master Mix, 0.5 µl of each primer, and 2 µl of genomic DNA. The protocol included an initial denaturation at 95 °C for 3 min, 24 cycles of denaturation at 94 °C for 30 s, annealing at 50 °C for 30 s, extension at 72 °C for 30 s, and a final extension at 72 °C for 3 min. After the amplification PCR, we combined duplicate samples and cleaned them using the Qiagen QIAquick PCR Purification Kit. We conducted a second PCR to add indexes to samples in duplicate from Sets A, B, and C of the Illumina Nextera XT Index Kit v2. The indexing PCR recipe included 12 µl of water, 5 µl of Qiagen AllTaq Master Mix, 0.5 µl of each primer, and 2 µl of the amplification PCR product. This protocol included an initial denaturation at 95 °C for 3 min, 8 cycles of denaturation at 95 °C for 30 s, annealing at 55 °C for 30 s, extension at 72 °C for 30 s, and a final extension at 72 °C for 5 min.

After the indexing PCR, we combined duplicate samples and quantified DNA concentrations using a Qubit 4 fluorometer. We diluted all samples to a concentration of 1 nMol and pooled samples into three distinct sequencing libraries. Each library contained positive and negative controls that went through both rounds of PCR. We paired-end sequenced (2 ×150 bp) the three pools of indexed samples on the Illumina iSeq 100 (Illumina, San Diego, CA, USA).

### Microbiome sequence processing

We used DADA2 [41] to process reads into amplicon sequence variants (ASVs) and aligned ASVs to the SILVA reference database [42] for taxonomy assignment. Aside from adjusting the forward read trimming step (150 bp), we used the default DADA2 pipeline. We used the *decontam* package [43] in R to bioinformatically remove contaminant sequences from all tick DNA samples. This procedure removes contaminant reads based on their prevalence in the negative controls. Application of *decontam* reduced the number of ASVs from 3,775 to 3,756. Next, we removed ASVs not classified as bacteria and 21 samples that did not yield sufficient reads for microbiome analysis, resulting in a final dataset of 259 tick samples (Supplementary Table S1). For analyses of alpha and beta diversity, we rarefied the dataset to the lowest sample read count of 2,500 reads; this rarefied dataset contained 3,235 ASVs (Supplementary Table S2). For analyses of relative abundance, we did not rarefy the dataset, but instead removed ASVs with less than 20 reads across all samples to minimize the effect of extremely rare ASVs [44]; this pruned dataset contained 2,016 ASVs (Supplementary Table S3).

### Statistical analyses

We conducted statistical analyses and produced figures in R [45]. All code used to analyze the microbiome sequencing data can be found in Supplementary File S1. To analyze beta diversity, we used principal coordinates analysis (PCoA) based on weighted and unweighted UniFrac distances [46]. We then conducted a nested PERMANOVA with 999 permutations to evaluate differences in the overall structure of microbial communities among regions, among sites within regions, and between sexes within sites. To analyze alpha diversity, we calculated ASV richness and the Shannon index for each tick. We then conducted a nested PERMANOVA with 999 permutations to evaluate differences in these alpha diversity metrics among regions, among sites within regions, and between sexes within sites. We used the *picante* package [47] to evaluate the phylogenetic alpha diversity within each tick by calculating two measures: Net Relatedness Index (NRI) and Nearest Taxon Index (NTI). Positive values for these metrics are evidence of phylogenetic clustering due to environmental filtering, while negative values are evidence of phylogenetic overdispersion due to competitive exclusion [48]. We tested the significance of each computed NRI and NTI with 999 random permutations.

To visualize relative abundance of bacterial genera by site, we used the *phyloseq* package [49] to create a stacked bar plot excluding ASVs not identified to the genus level. We statistically analyzed relative abundance with Microbiome Multivariable Associations with Linear Models (MaAsLin2) tests using the *MaAsLin2* package [50] and with Linear Discriminant Analysis Effect Size (LEfSe) tests using the *lefser* package [51]. We conducted all MaAsLin2 and LEfSe tests on aggregated genus-level read counts relativized per tick to evaluate taxonomic differences at the highest level of specificity attainable, as done by Duncan et al. [31]. Since region and sex were the most important explanatory variables in beta and alpha diversity analyses, we performed MaAsLin2 tests to investigate sex differences in tick microbiome by region (i.e., comparing western males to western females, comparing northern males to northern females, and comparing colony males to colony females). We then similarly used MaAsLin2 tests to investigate regional differences in tick microbiome by sex (i.e., comparing western males to northern males, and comparing western females to northern females). We performed all MaAsLin2 tests using a q-value threshold of 0.05 (Benjamini-Hochberg adjustment) to identify significant differentially abundant taxa. However, we only retained associations with coefficients >|0.01| to exclude differentially abundant taxa which are statistically significant but lack biological relevance due to their low effect size. We then used LEfSe analyses to further elucidate differentially abundant taxa between wild ticks. We performed an initial LEfSe analysis to identify differentially abundant taxa by sex while controlling for the effect of region, and a subsequent LEfSe analysis to identify differentially abundant taxa by region while controlling for the effect of sex. We performed all LEfSe tests using a p-value threshold of 0.05 to identify significant differentially abundant taxa.

We used PICRUSt2 [52] with default settings to infer the functional potential of the bacterial community from the ASV dataset with raw read counts used for relative abundance analysis. PICRUSt2 predicts gene family abundances for each ASV based on phylogenetic placement against reference genomes and then maps these predicted gene families to metabolic pathways in the MetaCyc database [53]. We further classified metabolic pathways into higher-order categories grouping functionally similar pathways. The PICRUSt2 output and MetaCyc classification can be found in Supplementary Table S4. We relativized this output per tick and aggregated the data into identified higher-order categories prior to analysis with MaAsLin2 and LEfSe to identify differentially abundant groups of metabolic pathways between sexes and geographic regions. Similar to the taxonomic compositional analyses, we performed three MaAsLin2 analyses investigating sex differences by region (i.e., comparing western males to western females, comparing northern males to northern females, and comparing colony males to colony females) and two MaAsLin2 analyses investigating regional differences by sex (i.e., comparing western males to northern males, and comparing western females to northern females). These analyses used a q-value threshold of 0.05 (Benjamini-Hochberg adjustment) to identify significant associations. We then used LEfSe analyses to further examine differentially abundant metabolic pathway groups, performing one analysis comparing sexes while controlling for the effect of region, and another comparing regions while controlling for the effect of sex. We performed all LEfSe tests using a p-value threshold of 0.05 to identify significant differentially abundant metabolic pathways.

We calculated pathogen prevalence as the proportion of sampled ticks at each region that were positive for each of three bacterial genera associated with tick-borne disease: *Borrelia*, *Rickettsia*, and *Ehrlichia*. We then conducted Fisher’s exact tests to analyze the association between region and infection status separately for each genus, and the association between sex and infection status separately for each region and each genus.

## Results

### Sequencing

We characterized the microbiome of 129 adult male and 130 adult female *Amblyomma americanum* ticks from two distant regions of range expansion and a colony. The sequencing yielded a total of 2,695,246 reads (average per sample: 10,406, range: 2,506 - 252,259) that passed quality control filters. These reads clustered into 2016 distinct ASVs which were assigned to 17 bacterial phyla, 25 classes, 62 orders, 97 families, and 215 genera. Genus abundances from the positive control samples can be found in Supplementary Figure S1.

### Beta diversity

**Fig. 2:**
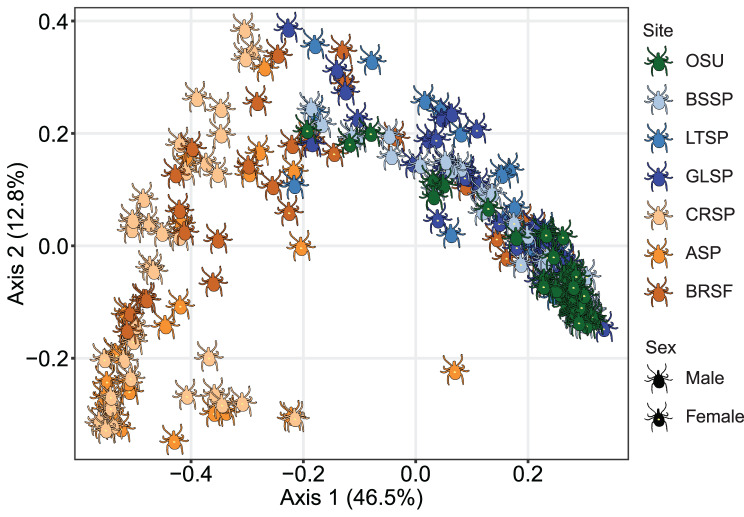
PCoA of beta diversity based on weighted UniFrac distances. Each tick icon represents one tick-associated bacterial community. OSU = Oklahoma State University Tick Rearing Facility. Blue shades indicate western sites: BSSP = Boiling Springs State Park, LTSP = Lake Thunderbird State Park, GLSP = Greenleaf State Park. Orange shades indicate northern sites: CRSP = Connetquot River State Park, ASP = Allaire State Park, BRSF = Bass River State Forest

The PCoA based on weighted UniFrac distances, which accounts for taxa abundances, showed significant clustering by region, site, and sex (Fig. 2). The first principal coordinate axis separates the OK ticks (including OSU colony ticks) from NY/NJ ticks; the second principal coordinate axis separates males from females. In the nested PERMANOVA that partitions sources of variation in microbiome beta diversity, region explained 27.3% of total variance; after accounting for region, site explained an additional 10.4%, and after accounting for both region and site, sex explained 14.1% of the remaining variance (all *p* < 0.001).

The PCoA based on unweighted UniFrac distances, which only accounts for taxon presence or absence, also showed significant clustering by region, site, and sex, although to a lower degree than the weighted analysis (Supplementary Figure S2). In the nested PERMANOVA, region explained 9.2% of total variance; after accounting for region, site explained an additional 7.4%, and after accounting for both region and site, sex explained 9.2% of the remaining variance (all *p* < 0.001).

### Alpha diversity

**Fig. 3:**
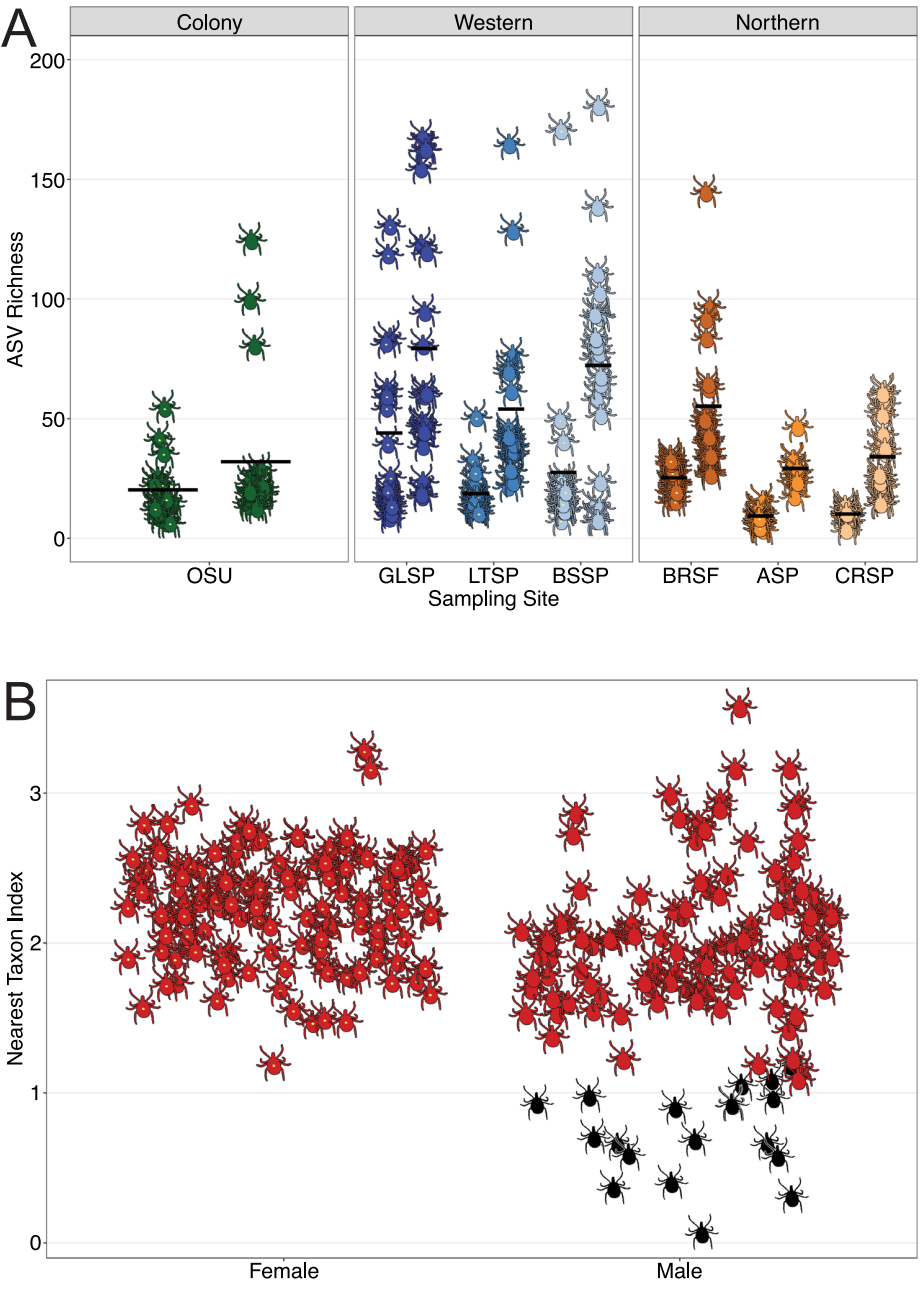
Alpha diversity of microbial communities in 259 *Amblyomma americanum* adults. **A**. ASV richness by sampling site; for each site, females are on the left and males on the right. Each tick icon represents one tick-associated bacterial community; horizontal bars represent group means. **B**. Nearest taxon index, a metric of phylogenetic alpha diversity. Each tick icon represents one tick-associated bacterial community. Red represents significant phylogenetic clustering (*p* < 0.05) and black represents non-significant values. Higher NTI values indicate stronger clustering near the tips of the phylogenetic tree

Similar to our analysis of beta diversity, ASV richness varied across regions, sites, and sexes (Fig. 3A). The nested PERMANOVA of ASV richness revealed that microbiome alpha diversity is significantly affected by region (R^2^ = 0.06, *p* < 0.001), site within each region (R^2^ = 0.11, *p* < 0.001), and sex within each site (R^2^ = 0.29, *p* < 0.001). Comparing regions, western sites in OK had significantly higher ASV richness than northern sites in NY/NJ (*p* = 0.002) and the OSU colony (*p* = 0.005). Comparing sexes within each site, ASV richness was significantly higher in males than in females at all western and all northern sites (all *p* < 0.022); ASV richness was also higher in colony males than in colony females, but the difference was not statistically significant (*p* = 0.153). The Shannon diversity index, which considers both richness and evenness, also varied across regions, sites, and sexes (Supplementary Figure S3A). The nested PERMANOVA of Shannon diversity revealed that microbiome alpha diversity is significantly affected by region (R^2^ = 0.05, *p* < 0.001), site within each region (R^2^ = 0.07, *p* < 0.001), and sex within each site (R^2^ = 0.20, *p* < 0.001). Comparing regions, the OSU colony had significantly lower Shannon diversity than northern sites in NY/NJ (*p* = 0.014) and western sites in OK (*p* = 0.004); there was no significant difference in Shannon diversity between western and northern regions (*p* = 0.065). Comparing sexes within each site, Shannon diversity was significantly higher in males than in females at all western and all northern sites (all *p* < 0.05); Shannon diversity was also higher in colony males than in colony females, but the difference was not statistically significant (*p* = 0.703). Altogether, these results indicate that geographic region, local ecology, and captivity influence the richness and evenness of the tick microbiome. Further, the effect of sex on tick microbiome diversity is more pronounced in wild ticks.

NTI and NRI, two distinct measures of phylogenetic alpha diversity within each tick, were positive for all 259 ticks. NTI values were significantly higher than expected by chance (*p* < 0.05) for all females and 85.3% of males (Fig. 3B). NRI values were significantly higher than expected by chance (*p* < 0.05) for 97.7% of females and 81.4% of males (Supplementary Figure S3B).

### Differentially abundant taxa

**Fig. 4:**
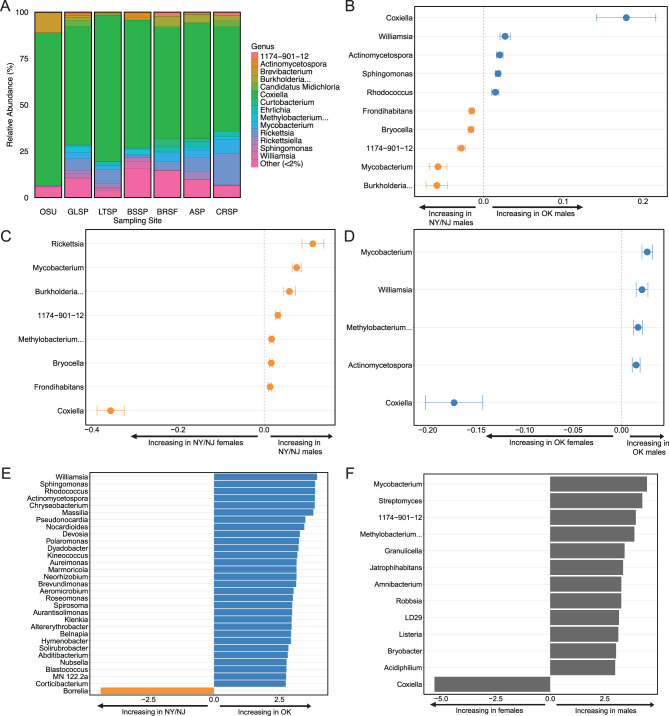
**A**. Relative abundance of bacterial genera at each site. **B**. MaAsLin2 analysis comparing western males to northern males. Positive values in blue indicate taxa more abundant in western males, and negative values in orange indicate taxa more abundant in northern males. **C**. MaAsLin2 analysis comparing northern males to northern females. Positive values indicate taxa more abundant in males, and negative values indicate taxa more abundant in females. **D**. MaAsLin2 analysis comparing western males to western females. Positive values indicate taxa more abundant in males, and negative values indicate taxa more abundant in females. **E**. LEfSe analysis comparing western ticks to northern ticks, controlling for the effect of sex. Positive values in blue indicate taxa more abundant in OK, and negative values in orange indicate taxa more abundant in NY/NJ. **F**. LEfSe analysis comparing wild males to wild females, controlling for the effect of region. Positive values indicate taxa more abundant in males, and negative values indicate taxa more abundant in females. MaAsLin2 graphs plot model coefficients along the x-axis, showing significant associations (*p* < 0.05) with coefficients >|0.01|; error bars depict the standard error of each association. LEfSe graphs plot log_10_ of the LDA score along the x-axis, showing significant associations (*p* < 0.05) with LDA scores >|2|

The bacterial genus *Coxiella* dominated the microbiome relative abundance at all sampling sites (Fig. 4A). The proportion of reads belonging to *Coxiella* at each site ranged between 56% to 80% (65% on average), with the highest proportion in OSU. Following *Coxiella*, *Brevibacterium* was highly abundant in the OSU colony ticks. This genus was much more abundant in the colony compared to any natural site and likely contributes to the reduced richness and evenness observed in the colony ticks.

We used MaAsLin2 to statistically compare differentially abundant ASVs, aggregated based on their genus-level classification, according to region and sex. The MaAsLin2 analysis investigating regional differences in males identified 10 significant taxa, of which 5 were more abundant in OK (*Coxiella*, *Williamsia, Actinomycetospora, Sphingomonas,* and *Rhodococcus*) and 5 were more abundant in NY/NJ (*Frondihabitans, Bryocella, 1174–901-12, Mycobacterium,* and *Burkholderia*) (Fig. 4B). The corresponding MaAsLin2 analysis investigating regional differences in females identified only two taxa, but both had an effect size coefficient below 0.01, causing the test to be excluded from graphical representation. The MaAsLin2 analysis comparing northern males to northern females identified 8 differentially abundant taxa with large effect sizes, of which *Coxiella* was the only taxon significantly more abundant in females (Fig. 4C). The MaAsLin2 analysis comparing western males to western females identified 5 differentially abundant taxa with large effect sizes, of which *Coxiella* again was the only taxon significantly more abundant in females (Fig. 4D). The MaAsLin2 analysis comparing colony males to colony females identified no differentially abundant taxa.

The LEfSe analysis of regional differences comparing western to northern ticks while eliminating the confounding sex differences in microbiome composition identified 31 differentially abundant taxa, of which *Borrelia* was the only taxon significantly more abundant in NY/NJ ticks (Fig. 4E). The LEfSe analysis of sex differences comparing wild male to wild female ticks while eliminating the confounding regional differences in microbiome composition identified 13 differentially abundant taxa, of which *Coxiella* was the only taxon significantly more abundant in female ticks (Fig. 4F).

### Functional metagenome

Results of the MaAsLin2 and LEfSe analyses of differentially abundant metabolic pathways are in Supplementary File S2. The MaAsLin2 analysis of the functional metagenome comparing western males to western females identified 65 differentially abundant pathway groups, 17 of which are more abundant in females. Focusing on the vitamin metabolism groups, four are more abundant in females and four are more abundant in males. The heme biosynthesis pathway is more abundant in females. The MaAsLin2 analysis comparing northern males to northern females also identified 65 differentially abundant pathway groups, 18 of which are more abundant in females. The same eight vitamin groups are abundant in female and male ticks; the heme biosynthesis pathway is more abundant in females. The MaAsLin2 analysis comparing colony males to colony females identified five differentially abundant pathway groups, all of which are more abundant in males and none of which are vitamin or heme pathways. The MaAsLin2 analysis of regional differences comparing western males to northern males identified 47 differentially abundant pathway groups, 22 of which are more abundant in western ticks. Five vitamin groups are more abundant in OK, while two are more abundant in NY/NJ. The heme biosynthesis pathway is more abundant in OK. The MaAsLin2 analysis comparing western females to northern females identified eight differentially abundant pathway groups, all of which are more abundant in western ticks and none of which are vitamin or heme pathways.

**Fig. 5:**
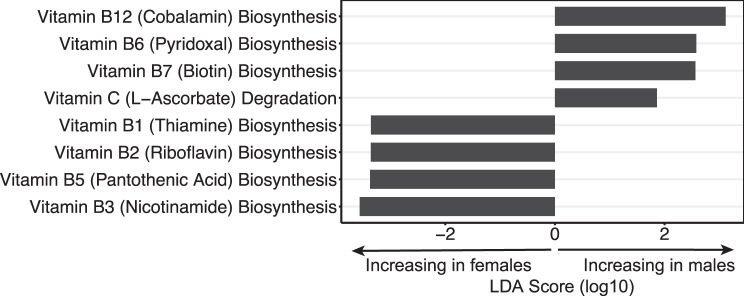
LEfSe analysis of differentially abundant vitamin metabolism pathways comparing wild males to wild females, controlling for the effect of region. Positive values indicate pathways more abundant in males, and negative values indicate pathways more abundant in females

The LEfSe analysis of sex differences comparing wild male to wild female ticks while eliminating confounding regional differences identified 58 differentially abundant pathway groups, 16 of which are more abundant in females. The same eight vitamin groups identified in both MaAsLin2 analyses are abundant in female and male ticks (Fig. 5). The heme biosynthesis pathway is more abundant in females, consistent with the MaAsLin2 analyses. The LEfSe analysis comparing western ticks to northern ticks while eliminating confounding sex differences identified two differentially abundant pathway groups, Protein Modification and Vitamin B6 Degradation, both of which are more abundant in OK.

### Prevalence of taxa associated with tick-borne pathogens

The 16S sequence data had limited taxonomic resolution, allowing us to identify bacterial genera, but not specific species or strains. The prevalence of ticks carrying bacterial genera containing species and strains typically associated with tick-borne diseases varied across regions and between sexes within a region (Table 1). *Borrelia* was detected only in northern sites, and its prevalence was significantly higher in the northern region compared to the western region (corrected odds ratio OR = 26.9, *p* < 0.001). *Rickettsia* was detected in all sites including the colony, and its prevalence was significantly higher in the northern region compared to the western region (OR = 3.2, *p* < 0.001). *Ehrlichia* was detected at high prevalence rates in all sites including the colony, and its prevalence was not significantly different in the northern region compared to the western region (OR = 1.1, *p* = 0.871).

**Table 1:** Percentage of ticks carrying bacteria belonging to three genera associated with tick-borne pathogens

Region	Sex	N	Borrelia	Rickettsia	Ehrlichia
Northern	M	49	10.2	77.6	22.4
	F	53	9.4	67.9	22.6
Western	M	60	0	56.7	26.7
	F	58	0	32.8	15.5
Colony	M	20	0	55.0	20.0
	F	19	0	10.5	5.3

Western males were more likely to carry *Rickettsia* than western females (OR = 2.66, *p* = 0.010), and colony males were almost 10 times more likely to carry *Rickettsia* than colony females (OR = 9.73, *p* = 0.006); there was no significant difference in *Rickettsia* prevalence between males and females in the northern region. There were no significant differences in any region in the prevalence of *Borrelia* or *Ehrlichia* between male and female ticks.

## Discussion

Our study is, to our knowledge, the largest tick microbiome study to date based on next-generation sequencing of the 16S rRNA bacterial gene, with a robust sample size of 259 independent bacterial communities associated with *Amblyomma americanum* sampled from six natural sites in two distant regions of the species range and from one laboratory-reared colony. Additionally, it is also the first study that focuses on evaluating both sexual and regional differences in the microbiome and functional metagenome of *A. americanum*, particularly in areas of recent range expansion.

### Geographic region and sex strongly influence alpha and beta diversity

We found that both geographic region and sex strongly influence alpha and beta diversity of the lone star tick microbiome. This was the case whether we analyzed alpha diversity by ASV richness or Shannon diversity index, and whether we analyzed beta diversity by weighted or unweighted UniFrac distances (Figs. 2–3, Supplementary Figures S2–S3). Interestingly, we observed higher alpha diversity in the western region than in the northern region. We also observed significant differences in alpha diversity among the three western sites separated by 194–229 km and among the three northern sites separated by 63–107 km. This finding shows that the external ecological context of a site influences the tick’s internal microbiome. For example, Allaire State Park (ASP) is only 63 km from Bass River State Forest (BRSF); yet, the microbiome of ticks in ASP which is characterized by a combination of mixed and deciduous forest differs significantly from the microbiome of ticks in BRSF which is characterized by pine barrens [2]. Several studies also found that geographic region and sex are significant predictors of alpha and beta diversity of tick microbiomes [30–32, 54]. However, Chicana et al. examined the microbiomes of three species of ticks in the western United States - *Ixodes pacificus*, *Dermacentor albipictus*, and *Dermacentor occidentalis* - and detected no significant geographic variation in microbial richness, diversity, or composition between ticks collected from two sites located approximately 64 km apart [15].

We observed that males uniformly had higher microbiome diversity than females at all natural sites, but not at the colony. These results concur with various other studies that found higher microbiome alpha diversity in male ticks of various ixodid genera including *Amblyomma* [54–57], *Dermacentor* [30, 31], *Ixodes* [32, 57–59], *Rhipicephalus* [60, 61], *Haemaphysalis* [62], and *Hyalomma* [63]. Thus, the pattern of males harboring more diverse microbiomes than females is widespread among tick taxa.

### Host environmental filtering governs bacterial community assembly, particularly in female ticks

The sex-based differences highlighted above are further demonstrated by analyses of NTI and NRI. NTI, which emphasizes terminal (tip-level) clustering, was significantly positive for all females and for the majority of males, suggesting that closely related bacterial taxa inhabit individual ticks, especially in females. NRI, which captures deeper phylogenetic relationships, was also significantly positive in most ticks, further supporting the inference of phylogenetic conservatism in microbiome assembly. The higher rate of significant phylogenetic clustering in females reflects more stable bacterial communities. Conversely, the lower rate of clustering in males reflects more variable or transient bacterial communities. These findings suggest sex-specific differences in the determinism and phylogenetic structure of tick microbiomes, with females showing stronger and more consistent patterns, possibly due to more stable associations with endosymbionts or differences in feeding behaviors and reproductive roles.

### Geographic region and sex strongly influence relative abundance of specific taxa

We found *Coxiella* to be very abundant and dominant over all other bacterial genera (Fig. 4A). This finding is not surprising because it is consistent with other studies that found *Coxiella* as the predominant endosymbiont of *A. americanum* [56, 65]. Not only was *Coxiella* highly abundant, it was also highly prevalent, detected in all ticks in the colony, all ticks in the western region, and all but 7 male ticks in the northern region. Thus, we found *Coxiella* in all laboratory-reared and field-collected *A. americanum* females and most field-collected males, consistent with the first report showing evidence that *Coxiella* may be an obligate endosymbiont [66].

Only male ticks had bacterial taxa that are differentially abundant between regions (Fig. 4B); female ticks had no taxa that are differentially abundant between regions. This novel finding further supports our earlier conclusion that the external environment (e.g. vertebrate hosts, soil, vegetation) affects the composition and relative abundance of male microbiomes, and that female microbiomes are more stable. The LEfSe analysis found a higher number of overabundant taxa in western sites than in northern sites (Fig. 4E). Interestingly, *Coxiella* is not one of these taxa identified in the regional LEfSe analysis. Since the LEfSe analysis investigated regional differences while controlling for the effect of sex, it identified taxa whose differential abundance in a region is consistent in both males and females. Hence, the overabundance of *Coxiella* in OK and the five taxa overabundant in NY/NJ identified in the MaAsLin2 regional analysis (Fig. 4B) are only differentially abundant in male ticks and not in female ticks. Additionally, our MaAsLin2 analyses excluded rare genera present in less than 10% of samples, while Lefse analyses did not; ten genera included in the LEfSe (e.g. *Borrelia*) are below that threshold. Together these MaAsLin2 and LEfSe analyses describe the regional differences in microbiome identified by the beta diversity analysis and concur with other tick microbiome studies which found a significant effect of region [31, 32, 54, 67]. However, no study to date has documented the interaction between sex and region described above.

In both regions of range expansion, *Coxiella* was the only genus overly overabundant in female ticks; male ticks featured a higher number of overly abundant taxa (Fig. 4C–D), consistent with the finding that males have higher alpha diversity than female ticks (Fig. 3A, Supplementary Figure S3A). Only two of the seven taxa (*Mycobacterium* and *Methylobacterium*) identified as overabundant in northern males were also identified as overabundant in western males, suggesting that while *Mycobacterium* and *Methylobacterium* may be uniquely suited to fill the space left by a reduced dominance of *Coxiella* in males, regional differences in microbiota also contribute to which other bacteria become abundant in males. For example, *Rickettsia* was the most differentially abundant genus only in northern males. Williams-Newkirk et al. also found that males contain more *Rickettsia* and females contain more *Coxiella* [56]. The LEfSe analysis investigating sex-based differences in wild ticks while controlling for the effect of region largely corroborates MaAsLin2 results, showing a high number of taxa overabundant in males and the overabundance of *Coxiella* in females (Fig. 4F). This sex-based pattern of *Coxiella* abundance is entirely absent in the colony ticks.

### Distinct taxonomic profiles of the microbiome lead to distinct functional profiles between the sexes

In analyses comparing males and females in both northern and western regions, males had a higher number of differentially abundant metabolic pathway groups, indicating that males possess a more functionally diverse microbiome. This mirrors the taxonomic patterns between sexes, the higher functional diversity in males being a product of higher microbial diversity. Further, the colony ticks showed few differentially abundant metabolic pathways between males and females, reflecting their similar taxonomic profiles and indicating that the artificial conditions of the laboratory-reared colony alter microbial functions observed in wild ticks.

Obligate blood-feeders like ticks are challenged with a hyper-specialized diet lacking in key nutrients such as carbohydrates, lipids, and vitamins [68]. B vitamins are largely absent in a blood-based diet and ticks are incapable of synthesizing them de novo. However, ticks counteract this nutritional deficiency by forming close associations with vitamin-synthesizing bacteria. *Coxiella-*like endosymbionts (CLEs) are the most widespread vitamin B-producing endosymbiont in ticks, being associated with approximately two-thirds of tick species [68]. These endosymbionts typically dominate the microbiomes of their hosts and have been shown to be crucial for tick fitness at all life stages in multiple species [66, 69–71]. CLEs in *A. americanum* possess complete or partial metabolic pathways for the biosynthesis of B vitamins [11, 72].

We found four vitamin pathways are abundant in females and four in males (Fig. 5). This pattern was consistent in both regions, suggesting sex-based vitamin requirements regardless of external environment. The four pathways abundant in females are for the biosynthesis of vitamins B1, B2, B3, and B5, all of which are pathways linked to the *Coxiella* symbiont [72], which is abundant in female ticks (Fig. 4F). The four pathways abundant in males are for the biosynthesis of vitamins B6, B7, and B12 and the degradation of vitamin C; only two of these vitamins (B6 and B7) are attributed to *Coxiella* [72]. The higher number of *Coxiella-*associated pathways in female ticks suggests that females depend on *Coxiella* more than males. Other studies have found higher abundances of endosymbionts in ovaries than in testes and demonstrated that elimination of key endosymbionts impairs tick development to a higher degree in females than in males [70, 73].

Ticks also lack the ability to synthesize or degrade heme, despite it being both necessary for important functions, such as oxidative phosphorylation, and toxic in excess quantities due to its reactivity and role in inducing oxidative stress [74]. After hard ticks engorge themselves with blood and digest the hemoglobin therein, they must contend with an overabundance of free heme, which is cytotoxic. We did not identify heme degradation pathways in any bacteria in our study, suggesting that lone star ticks manage heme using other means that do not include microbes, such as sequestering it in hemosomes or with heme-binding proteins [75]. However, we identified six pathways related to heme biosynthesis and the pathway group was ubiquitous across our study. The heme biosynthesis pathway group was more abundant in females in both regions of wild ticks, but was not differentially abundant in colony females. Although ticks generally acquire heme in copious amounts directly from the bloodmeal, the differential abundance of heme biosynthesis pathways in wild females suggests that bacteria play a role in heme metabolism and potentially supplement their hosts with this essential cofactor [68]. This phenomenon may be affected by laboratory conditions which reduce sex-based differences in the microbiome of colony ticks.

These results should be interpreted as predicted metagenomic potential rather than confirmed functional capacity since our approach infers function indirectly, rather than measuring genes directly. Although PICRUSt2 enables a useful approximation of functional potential, it relies on the assumption that closely related taxa share similar genomic content. Given that our input sequences cover only the V4 region of the 16S rRNA gene, these predictions are necessarily coarse. Therefore, the inferred metabolic pathways we identified should be validated using shotgun metagenomics or long-read amplicon sequencing approaches.

### Geographic region and sex influence pathogen prevalence

The number and abundance of pathogenic bacteria in any individual tick are actually small compared to the full diversity of bacterial species and their abundances. Although our sequencing data cannot discriminate between pathogenic and non-pathogenic species or strains of *Borrelia* and *Rickettsia*, we found significant regional and sex-based differences in the prevalence of these two genera. Most *Borrelia* present in the lone star tick are from the species *B. lonestari*. We detected *Borrelia* only in northern sites at low prevalence (7% in site BRSF, 13% in site ASP, and 9% in site CRSP). An early survey of *B. lonestari* across the range of lone star tick found low prevalence rates: 0% in OK, 0.42% in NY, and 9.1% in NJ [76]. In fact, 54 surveys involving more than 52,000 lone star ticks have revealed a low prevalence of *B. lonestari* and scarce *B. burgdorferi* [77]. A more recent survey detected *B. lonestari* in 6 out of 522 (1.1%) adult lone star ticks collected in OK [78].

Most *Rickettsia* present in the lone star tick are from the species *R. amblyommatis*. We detected *Rickettsia* ASVs at a higher prevalence in the northern region compared to the western region. Similarly, Mixson et al. reported higher prevalence of *Rickettsia* in adult *A. americanum* collected from NY/NJ sites (34.6%) compared to those collected from OK (11.8%) [76]. We also observed adult males were 2.66 times more likely to carry *Rickettsia* than adult females in OK. This is contrary to an early survey that found no significant differences in *Rickettsia* prevalence between male and female *A. americanum* ticks across its range [76], and also a recent survey that focused on ticks in OK [78]. The much higher prevalence of *Rickettsia* we observed in colony males compared to colony females merits further investigation.

Most *Ehrlichia* present in the lone star tick are from the species *E. chaffeensis* and *E. ewingii*. Our observed prevalences of *Ehrlichia* in field-collected populations (22.5% in the northern region and 21.2% in the western region) are the highest reported to date. The highest site prevalence was at LTSP with 14/40 (35%) ticks infected with *Ehrlichia*. Mixson et al. reported *E. chaffeensis* prevalences across 9 states ranging from 0% to 13.2%; only one local site out of 29 sampled sites had a prevalence of *E. chaffeensis* that exceeded 20% [76]. In the same study, Mixson et al. reported *E. ewingii* prevalences ranging from 0% to 18.6%. Overall, these findings highlight geographic hotspots or temporal outbursts that exacerbate *Ehrlichia* transmission pressure, warranting further research and surveillance.

## Conclusions

Both sex and geographic region affect various aspects of the microbiome of *A. americanum*: alpha and beta diversity, relative abundance of bacterial taxa, functional diversity, and presence of pathogens. The relationship between microbial diversity, genomic diversity, and pathogen load is undergoing further investigation in our laboratory. Other avenues of future research should aim to elucidate the role of microbes within ticks in modulating oxidative stress from consuming heme-rich blood [79], climatic stress experienced at the periphery of their species range [80], or starvation stress from long periods of fasting [81]. Discovering the mechanisms through which microbes help ticks can make dysbiosis a potential strategy for tick control.

## Electronic supplementary material

Below is the link to the electronic supplementary material.

Supplementary Material 1

Supplementary Material 2

Supplementary Material 3

Supplementary Material 4

Supplementary Material 5

## Data Availability

The datasets generated and analyzed during the current study are available in the NCBI Sequence Read Archive, accession IDs PRJNA1277795 and PRJNA1278003, and are included in this published article’s supplementary information files.
