# Supplementary material for: Sexual and regional differences in the microbiome and functional metagenome of the lone star tick, Amblyomma americanum
Source: Anim Microbiome. 2025 Dec 6;7:127. doi: 10.1186/s42523-025-00498-6 (PMC12729126; doi:10.1186/s42523-025-00498-6)
Supplement: Supplementary file 3 — Supplementary Material 3 [file 42523_2025_498_MOESM3_ESM.pdf]

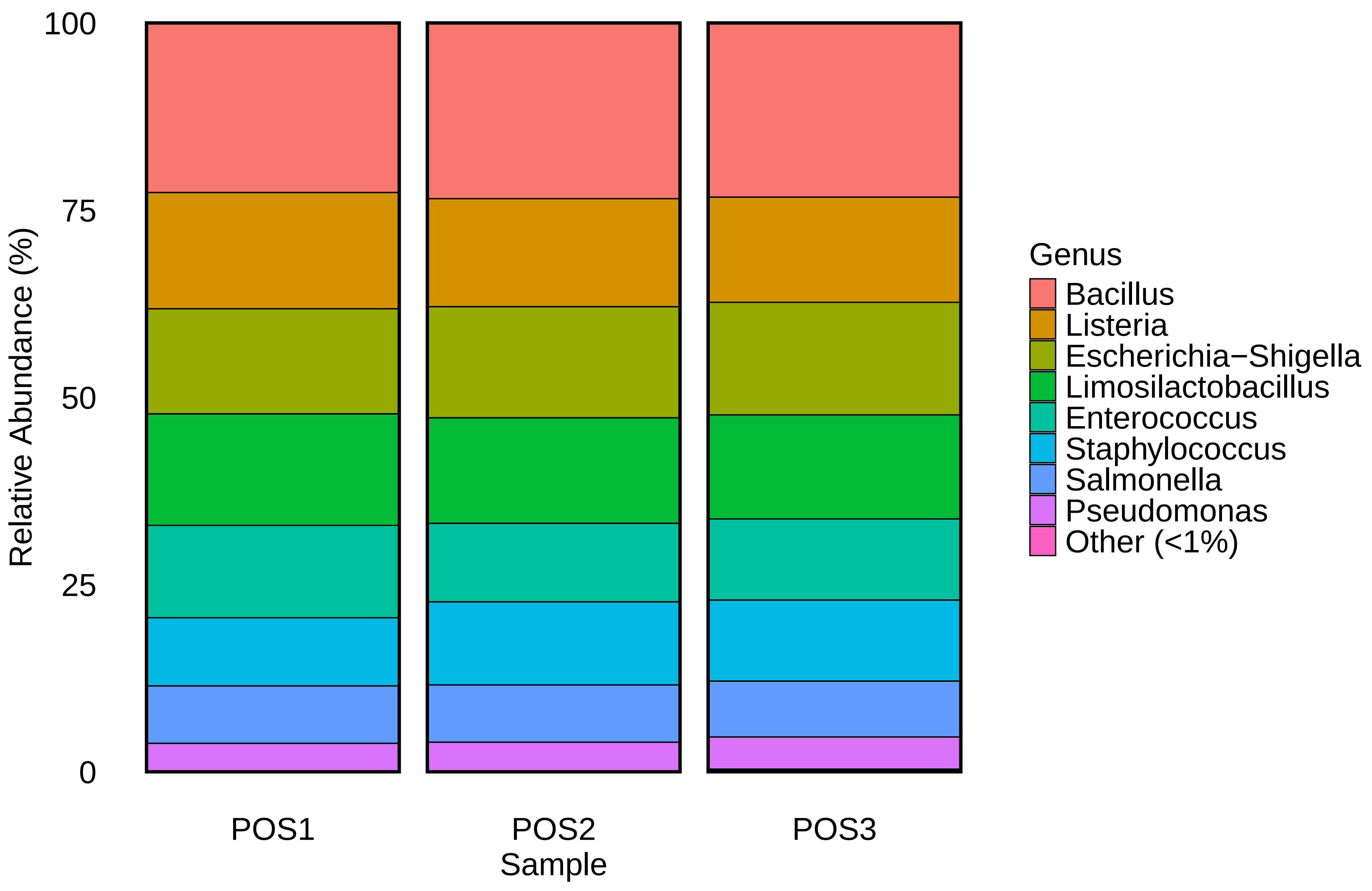

Relative Abundance (%)
100
75
50
25
0
POS1
POS2
POS3
Sample
Genus
Bacillus
Listeria
Escherichia−Shigella
Limosilactobacillus
Enterococcus
Staphylococcus
Salmonella
Pseudomonas
Other (<1%)
