# Supplementary figures and images for: Sexual and regional differences in the microbiome and functional metagenome of the lone star tick, Amblyomma americanum
Source: Anim Microbiome. 2025 Dec 6;7:127. doi: 10.1186/s42523-025-00498-6 (PMC12729126; doi:10.1186/s42523-025-00498-6)

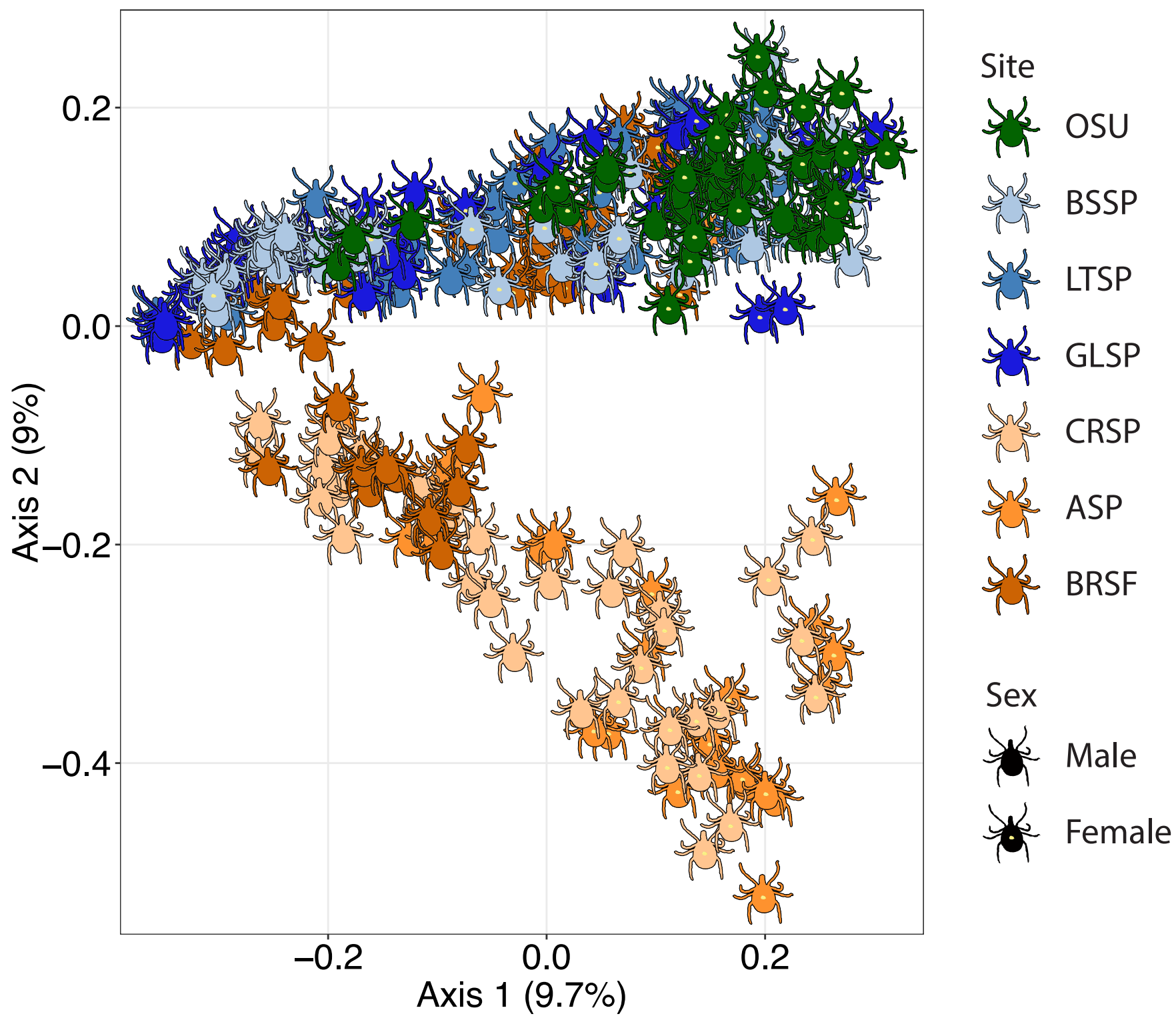

Supplement: Supplementary file 4 — Supplementary Material 4 [file 42523_2025_498_MOESM4_ESM.pdf]

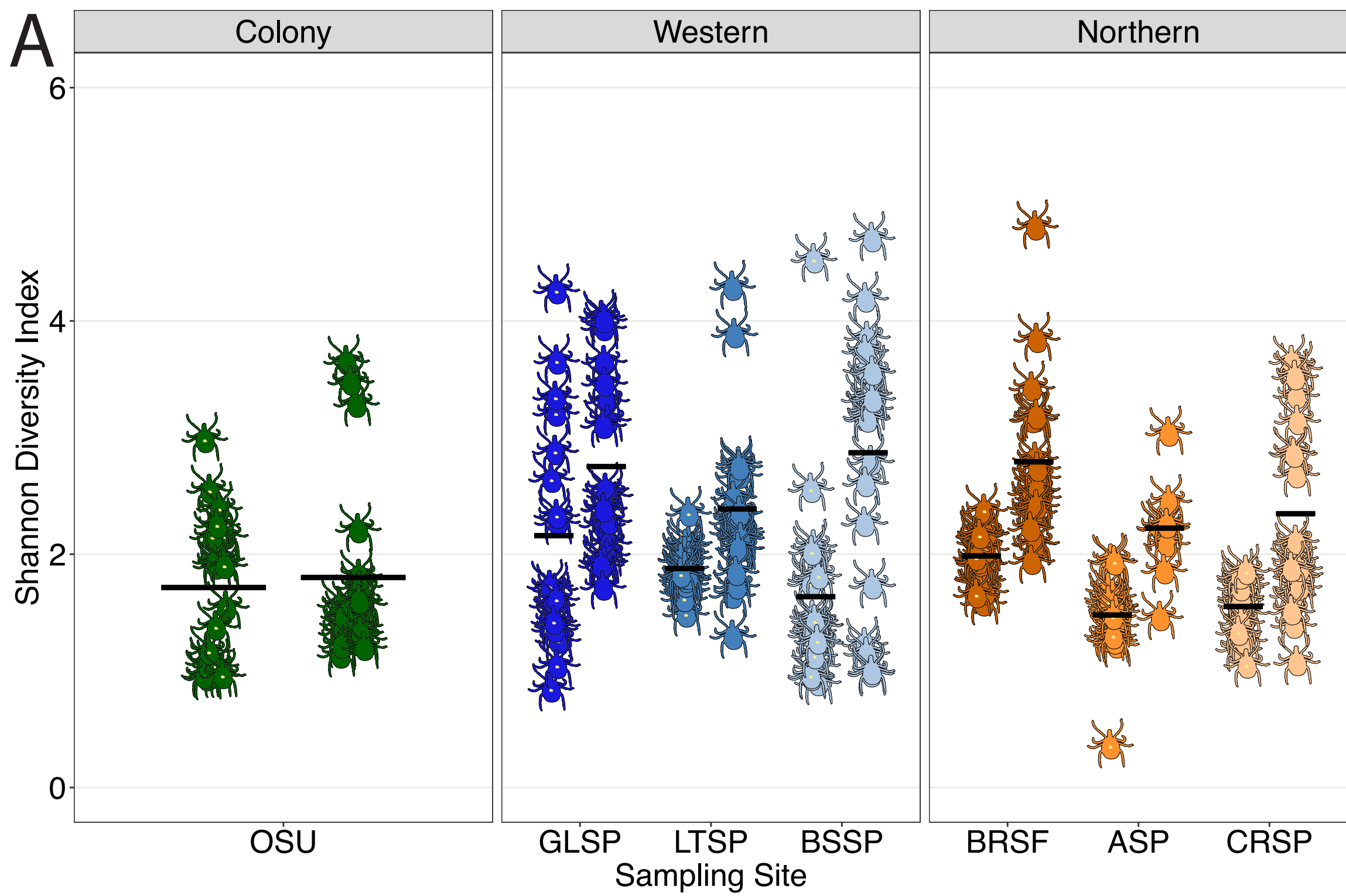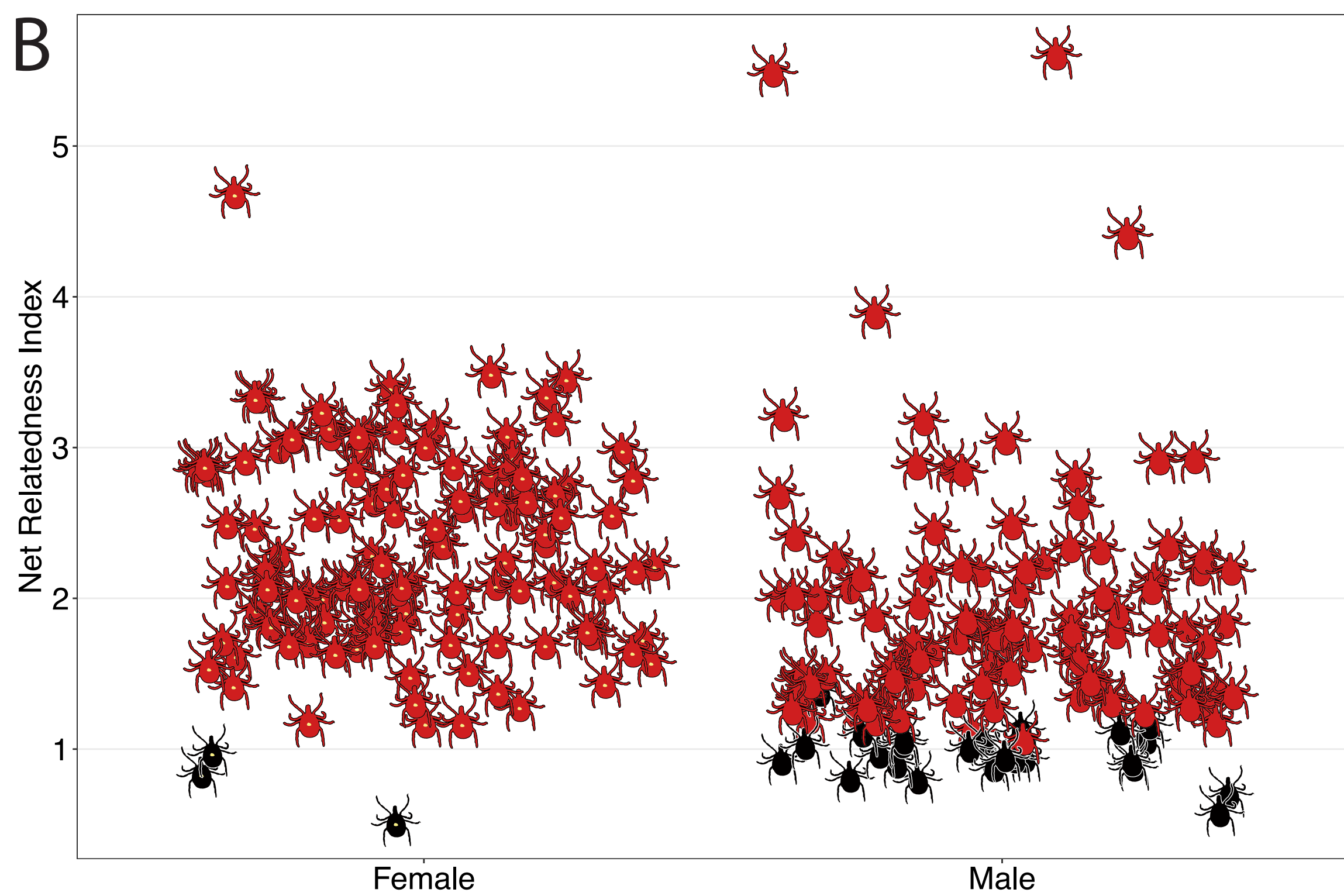

Supplement: Supplementary file 5 — Supplementary Material 5 [file 42523_2025_498_MOESM5_ESM.pdf]
